# DDRI-9: a novel DNA damage response inhibitor that blocks mitotic progression

**DOI:** 10.18632/oncotarget.7135

**Published:** 2016-02-02

**Authors:** Dong Wha Jun, Mihwa Hwang, Yun-Hee Kim, Kyung-Tae Kim, Sunshin Kim, Chang-Hun Lee

**Affiliations:** ^1^ New Experimental Therapeutics Branch, Division of Convergence Technology, National Cancer Center, Goyang, Gyeonggi, Korea; ^2^ Cancer Cell and Molecular Biology Branch, Division of Cancer Biology, National Cancer Center, Goyang, Gyeonggi, Korea; ^3^ Molecular Imaging and Therapy Branch, Division of Convergence Technology, National Cancer Center, Goyang, Gyeonggi, Korea; ^4^ Molecular Epidemiology Branch, Division of Cancer Epidemiology and Prevention, National Cancer Center, Goyang, Gyeonggi, Korea; ^5^ System Cancer Science, Graduate School of Cancer Science and Policy, National Cancer Center, Goyang, Gyeonggi, Korea

**Keywords:** DNA damage response, anticancer drugs, mitotic inhibitors, γH2AX, DNA repair-related proteins

## Abstract

The DNA damage response (DDR) is an emerging target for cancer therapy. By modulating the DDR, including DNA repair and cell cycle arrest, the efficacy of anticancer drugs can be enhanced and side effects reduced. We previously screened a chemical library and identified novel DDR inhibitors including DNA damage response inhibitor-9 (DDRI-9; 1H-Purine-2,6-dione,7-[(4-fluorophenyl)methyl]-3,7-dihydro-3-methyl-8-nitro). In this study, we characterized DDRI-9 activity and found that it inhibited phosphorylated histone variant H2AX foci formation upon DNA damage, delayed DNA repair, and enhanced the cytotoxicity of etoposide and ionizing radiation. It also reduced the foci formation of DNA repair-related proteins, including the protein kinase ataxia-telangiectasia mutated, DNA-dependent protein kinase, breast cancer type 1 susceptibility protein, and p53-binding protein 1, but excluding mediator of DNA damage checkpoint protein 1. Cell cycle analysis revealed that DDRI-9 blocked mitotic progression. Like other mitotic inhibitors, DDRI-9 treatment resulted in the accumulation of mitotic protein and induced cell death. Thus, DDRI-9 may affect both DDR signal amplification and mitotic progression. This study suggests that DDRI-9 is a good lead molecule for the development of anticancer drugs.

## INTRODUCTION

The maintenance of genomic integrity is essential for survival. Thus, the DNA damage response (DDR), which protects the genome from various insults, is highly conserved and complex. Cell cycle arrest mechanisms and DNA repair pathways are activated in response to DNA damage. If the damage is too severe, cells harboring the damaged DNA are removed from the organism through the induction of cell death. The cellular responses to DNA damage, which include DNA repair pathway activation, cell cycle arrest, and cell death induction, are collectively called the DDR [1, 2]. The DDR is important in the field of cancer therapy because both chemotherapy and radiotherapy are based on DNA damage-induced tumor cell death. However, activation of the DDR after treatment with chemotherapeutic DNA-damaging agents or radiation may promote tumor cell survival rather than induce cell death, which reduces the effectiveness of these treatments. This suggests inhibition of the DDR (cell cycle arrest and DNA repair) could increase the efficacy of conventional DNA-damaging agents.

Several small molecules that inhibit protein kinases involved in the DDR have been developed and characterized as chemosensitizers that could potentiate the efficacy of conventional cancer therapies [3-7]. For example, KU55933, a specific inhibitor of the protein kinase ataxia-telangiectasia mutated (ATM), which is a key player in the signaling pathways that are activated following DNA double-strand breaks (DSBs), enhances the cytotoxicity of ionizing radiation (IR) and DNA-damaging anticancer drugs (e.g., etoposide [ETO], doxorubicin, and camptothecin) [8]. Other small molecules, including UCN01, XL-844, AZD7762 and PF-0047736, which are inhibitors of the cell cycle checkpoint kinases 1 and 2 (the gatekeepers of cell cycle progression), have been developed as chemosensitizers and evaluated in several clinical trials [4, 9-11]. In addition to sensitization-based strategies, targeting the DDR in specific tumor cells is highly effective. A specific inhibitor of poly(ADP-ribose) polymerase (PARP), important for single-strand break (SSB) repair, can selectively kill tumor cells carrying BRCA mutations but not normal cells [12-14]. Thus, the application of inhibitors that target the DDR is a promising therapeutic strategy.

Several chemotherapeutic agents, including bleomycin, ETO, doxorubicin and camptothecin, as well as gamma irradiation, all induce DSBs, one of the most severe forms of DNA damage. The DSBs induce activation of ATM, which phosphorylates several downstream substrates. One of the initial targets of ATM is histone variant H2AX, which is phosphorylated on serine 139 at DSBs. Phosphorylated H2AX (γH2AX) recruits DDR proteins to DSBs to elicit DNA repair [15, 16]. One of DDR proteins is mediator of DNA-damage checkpoint protein 1 (MDC1), which is phosphorylated by ATM and binds γH2AX. MDC1 may serve as a platform for the recruitment of other DDR proteins and ATM-dependent amplification in response to DNA damage [17, 18]. It also regulates the intra-S-phase checkpoint, G2/M checkpoint, and mitotic progression [19-21]. Several studies have shown that DDR core proteins (e.g., ATM, p53-binding protein 1 [53BP1], and MDC1) are involved in both DNA repair and the regulation of mitotic progression, suggesting the processes are closely linked [22, 23]. Like the DDR, the mitotic phase of tumor cells has become a target for cancer therapy. Antimitotic drugs such as taxol, which interferes with microtubule dynamics, cause mitotic arrest and cell death [24].

We previously exploited the relationship between γH2AX foci formation and the extent of DNA damage to develop a quantitative cell-based high content screening method to identify chemical inhibitors of the DDR. Using this method, we screened a chemical library and identified several inhibitors of γH2AX foci formation [25]. In this study, we characterized DNA damage response inhibitor-9 (DDRI-9; 1H-Purine-2,6-dione,7-[(4-fluorophenyl)methyl]-3,7-dihydro-3-methyl-8-nitro) and found that it inhibits γH2AX foci formation upon DNA damage and blocks mitotic progression.

## RESULTS

### DDRI-9 inhibits γH2AX foci formation

**Figure 1:**
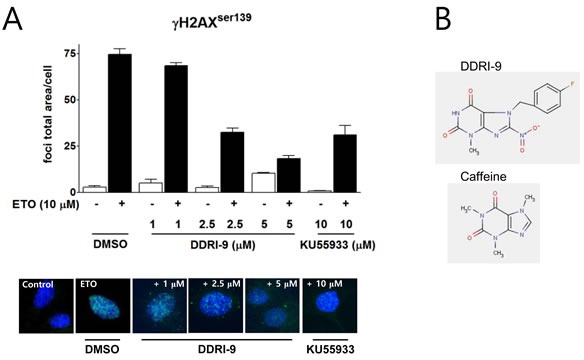
DDRI-9 inhibits γH2AX foci formation **A.** U2OS cells were pre-incubated with DMSO, DDRI-9, or KU55933 at the indicated concentrations for 1 h and then exposed to 10 μM ETO for 1 h. After incubation, the cells were fixed and processed for γH2AX immunofluorescence. The γH2AX foci were analyzed with an IN Cell Analyzer. The graphs and values represent the means ± SEM from three independent experiments. Representative images from three independent experiments are shown. **B.** The chemical structures of DDRI-9 and caffeine.

**Figure 2:**
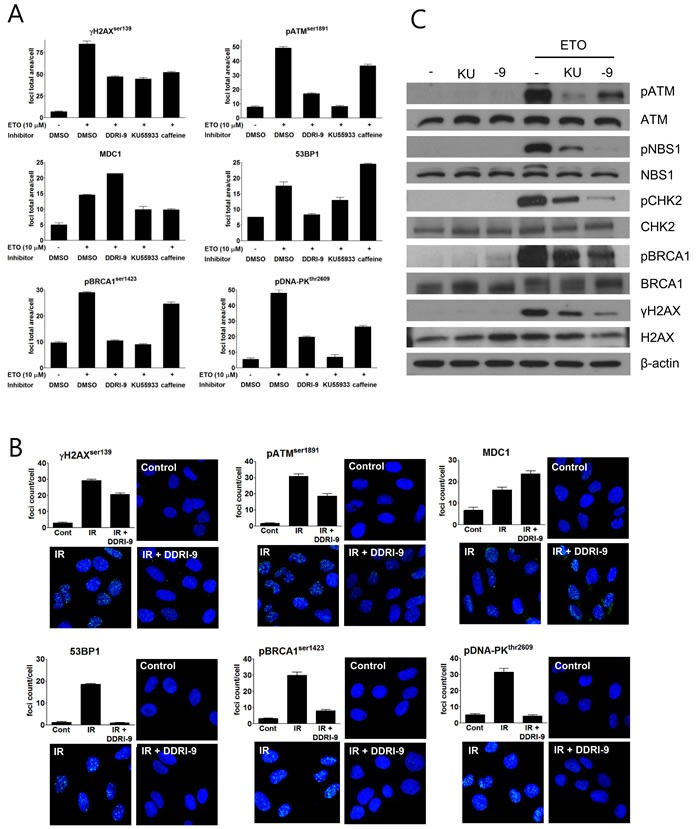
DDRI-9 inhibits the DDR signaling pathway **A.** U2OS cells were pre-incubated with DMSO, 2.5 μM DDRI-9, 10 μM KU55933, or 10 mM caffeine for 1 h and then exposed to 10 μM ETO for 1 h. After incubation, the cells were fixed and processed for immunofluorescence with the indicated antibodies. Foci were analyzed with an IN Cell Analyzer. Representative graphs and values from three independent experiments are shown. The values represent the means ± SEM. **B.** U2OS cells were pre-incubated with 2.5 μM DDRI-9 for 1 h and then exposed to 3 Gy IR. One hour after incubation, the cells were fixed and processed for immunofluorescence with the indicated antibodies, and images from 10 fields per test were collected using confocal microscopy and analyzed. Representative graphs and images from three independent experiments are shown. The values represent the means ± SEM. **C.** Protein extracts from U2OS cells treated as described in Figure 2A were analyzed by Western blotting using the indicated antibodies. KU, KU55933; -9, DDRI-9.

We previously established a high content screening method based on quantitation of γH2AX foci to identify small molecule inhibitors of the DDR and screened a chemical library (provided by the Korea Chemical Bank) containing 6,800 compounds. In an initial screen, 11 chemicals were identified that inhibited ETO-induced γH2AX foci formation. Among these compounds, DDRI-9 was the most potent inhibitor of DDR [25]. We examined whether DDRI-9 could inhibit γH2AX foci formation in a dose-dependent manner (Figure 1A). U2OS cells were pretreated with DDRI-9 for 1 h, exposed to 10 μM ETO for 1 h, fixed, and then immunostained with an anti-γH2AX antibody. In the presence of 1 μM, 2.5 μM, and 5 μM DDRI-9, the γH2AX foci total area per cell decreased from 74.51 ± 3.10 to 68.43 ± 1.67, 32.48 ± 2.23, and 18.30 ± 1.54, respectively. After treatment with 10 μM KU55933, a specific inhibitor of ATM that phosphorylates H2AX, the γH2AX foci total area per cell decreased from 74.51 ± 3.10 to 31.11 ± 5.08, similar to the effect of 2.5 μM DDRI-9. We confirmed that DDRI-9 inhibited γH2AX foci formation after ETO treatment at a lower concentration than KU55933. We also observed that DDRI-9 inhibited ETO-induced γH2AX foci formation in HeLa and MDA-MB-231 cells (Supplementary Figure 1). The chemical structure of DDRI-9 is similar to that of caffeine (Figure 1B). Because caffeine is an inhibitor of ATM and ataxia-telangiectasia and Rad3-related (ATR) kinase [26], we compared the inhibitory activity of caffeine with that of DDRI-9. Caffeine inhibited ETO-induced γH2AX foci formation at higher concentrations than DDRI-9 (Figure 2A).

### DDRI-9 alters recruitment of DDR-related proteins onto DNA damage sites and inhibits ATM-mediated DDR signaling pathways

In response to DSBs, autophosphorylated, active ATM phosphorylates H2AX at sites of DNA damage. Several DDR-related proteins such as MDC1, 53BP1, breast cancer type 1 susceptibility protein (BRCA1), and DNA-dependent protein kinase (DNA-PK) are then recruited to the DSBs. To characterize the inhibitory effects of DDRI-9 on DDR, we examined whether it affected recruitment of DDR-related proteins onto DNA damage sites. Following treatment of U2OS cells with ETO in the presence or absence of DDRI-9, we observed foci formation of DDR-related proteins (γH2AX, ATM, MDC1, 53BP1, BRCA1, and DNA-PK). In the presence of DDRI-9, the extent of γH2AX, ATM, 53BP1, BRCA1, and DNA-PK foci formation decreased as expected, but MDC1 foci formation increased (Figure 2A). We then evaluated the activities of KU55933 and caffeine, which are inhibitors of the DDR. Both chemicals inhibited foci formation of DDR-related proteins including MDC1. DDRI-9 treatment also increased MDC1 foci formation after ETO treatment in HeLa and MDA-MB-231 cells (Supplementary Figure 1). By confocal microscope analysis, DDRI-9 inhibited γH2AX, ATM, 53BP1, BRCA1, and DNA-PK1 foci formation in response to DSBs induced by ionizing radiation (IR). However, the MDC1 foci count per cell increased in the presence of DDRI-9 after IR (Figure 2B). Thus, DDRI-9 altered DDR-related protein foci formation. In response to DSBs, DDRI-9 inhibited the recruitment of most DDR-related proteins (γH2AX, ATM, 53BP1, BRCA1, and DNA-PK1) to DNA damage sites. However, MDC1 recruitment increased.

We also examined whether DDRI-9 inhibited ATM-dependent DDR signaling. Indeed, DDRI-9 inhibited phosphorylation of ATM, Nijmegen breakage syndrome gene (NBS1), BRCA1, checkpoint kinase 2 (CHK2), and H2AX in DSB-induced ATM signaling (Figure 2C).

### DDRI-9 inhibits DNA repair and ETO-induced cell cycle arrest

**Figure 3:**
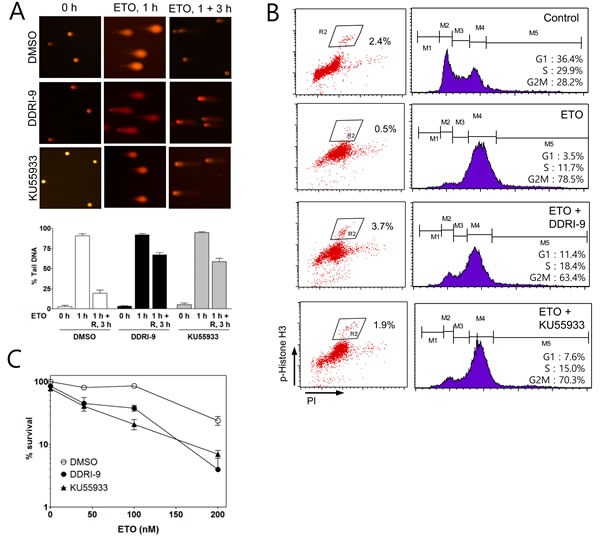
DDRI-9 inhibits DNA repair and cell cycle arrest **A.** U2OS cells were pretreated with DMSO, 2.5 μM DDRI-9, or 10 μM KU55933 for 1 h and then exposed to 10 μM etoposide for 1 h, followed by incubation in ETO-free medium in the presence of DMSO, 2.5 μM DDRI-9, or 10 μM KU55933 for 3 h. A comet assay was performed to detect tail DNA. The percentage of DNA in the tail is indicated on the y-axis. The values represent the means ± SEM. **B.** U2OS cells were pretreated with DMSO, 2.5 μM DDRI-9, or 10 μM KU55933 for 1 h and then incubated with 2 μM ETO for 24 h. After incubation, the cells were fixed and processed for immunofluorescence with an anti-phospho-histone H3 antibody and PI, followed by flow cytometry. **C.** U2OS cells were pretreated with 0.1 μM DDRI-9 or 5 μM KU55933 for 1 h and then incubated with etoposide at the indicated concentrations for 48 h, followed by incubation in ETO- and chemical-free medium until colony formation was observed. Survival was measured by counting colonies. Representative graphs from three independent experiments are shown. The values represent the means ± SEM.

We next examined whether DDRI-9 could disturb DNA repair similar to KU55933. An alkaline comet assay was performed to determine the extent of damaged DNA (extent of tail DNA) after ETO treatment in the presence and absence of DDRI-9. In the presence of vehicle dimethyl sulfoxide (DMSO), the extent of tail DNA was maximal 1 h after ETO treatment and decreased to the basal level 3 h after ETO removal from the culture medium. However, in the presence of DDRI-9, the extent of tail DNA remained at high levels 3 h after ETO removal, indicative of inefficient DNA damage repair. KU55933-treated cells showed a pattern of tail DNA after ETO removal that was similar to that observed with DDRI-9 treatment (Figure 3A). These data suggested that DDRI-9 inhibited DNA damage repair processes similar to KU55933.

Because several DDR inhibitors are known to interfere with DSB-induced cell cycle arrest, we examined whether DDRI-9 could disturb ETO-induced cell cycle arrest at the G2/M boundary. We performed cell cycle analysis based on combined staining with an anti-phospho-histone H3 (Ser10) antibody as a marker of mitosis. Treatment of U2OS cells with 2 μM ETO caused G2/M cell cycle arrest resulting in a reduced proportion of phospho-histone H3-positive cells. However, when cells were treated with ETO in combination with DDRI-9 or KU55933, G2/M arrest was disturbed and the number of phospho-histone H3-positive cells increased (Figure 3B). These data indicated that DDRI-9 and KU55933 blocked ETO-induced cell cycle arrest at G2.

Because several studies have suggested that DNA repair inhibitors including KU55933 could enhance the cytotoxicity of anticancer drugs, we tested whether DDRI-9 could sensitize tumor cells to ETO. We performed a clonogenic assay to measure U2OS cell viability after ETO treatment in combination with DDRI-9, KU55933, or DMSO as a vehicle control (Figure 3C). We determined that U2OS cells were more sensitive to ETO in the presence DDRI-9 and KU55933, and DDRI-9 enhanced the cytotoxicity of ETO in HeLa cells (Supplementary Figure 2A). Additionally, DDRI-9 rendered U2OS and HeLa cells more sensitive to IR (Supplementary Figure 2B). These data indicated that DDRI-9 increased the sensitivity of various tumor cells to anticancer drugs, similar to the effects of inhibitors of DNA damage repair.

### DDRI-9 blocks mitotic progression

**Figure 4:**
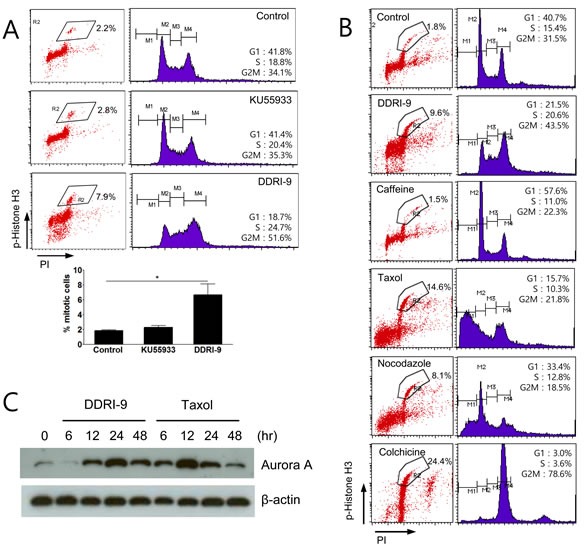
DDRI-9 blocks mitotic progression **A.** U2OS cells were treated with 10 μM KU55933 and 2.5 μM DDRI-9 for 24 h. After incubation, the cells were fixed and processed for immunofluorescence with anti-phospho-histone H3 antibody and PI, followed by flow cytometry analysis. Representative dot plots and histograms are shown. The graphs and values (below) represent the means ± SEM from three independent experiments (Student's *t*-test, (*) *P* < 0.05). **B.** U2OS cells were treated with 2.5 μM DDRI-9, 4 mM caffeine, 100 nM taxol, 200 nM nocodazole, and 100 ng/mL colchicine for 24 h and analyzed as described in Figure 4A. **C.** Protein extracts from U2OS cells treated with 2.5 μM DDRI-9 and 100 nM taxol for the indicated times were analyzed by Western blotting using specific antibodies.

Because treatment with ETO and either DDRI-9 or KU55933 increased phospho-histone H3-positive cells (Figure 3B), we examined whether DDRI-9 or KU55933 alone could affect the percentage of these cells. Although KU55933 treatment alone had no effect, DDRI-9 treatment alone increased the proportion of phospho-histone H3-positive cells (Figure 4A). Since the accumulation of phospho-histone H3-positive cells is indicative of defects in mitotic progression, we determined whether DDRI-9 could block mitotic progression, similar to the actions of taxol, nocodazole, and colchicine. Flow cytometric data demonstrated that DDRI-9 increased the percentage of phospho-histone H3-positive cells to a similar extent as these other antimitotic drugs. In contrast, caffeine did not block mitotic progression, even though it has a similar chemical structure to DDRI-9, (Figure 4B).

In addition, we found that DDRI-9 increased the percentage of phospho-histone H3-positive cells in other cell lines (HeLa, HCT116, MDA-MB-231, and Jurkat) (Supplementary Figure 3). Similar to taxol, DDRI-9 treatment increased the levels of the mitotic kinase Aurora A (Figure 4C). These data indicated that DDRI-9 blocked mitotic progression. Because DDRI-9 was identified as a DDR inhibitor, we examined whether taxol and nocodazole also inhibited DDR. However, neither chemical prevented DDR-related protein foci formation following treatment with ETO (Supplementary Figure 4). Taken together, these data indicate that DDRI-9 inhibited both DDR and mitotic progression, activities that are distinct from those of other mitotic and DDR inhibitors.

### DDRI-9 induces cell death through apoptosis

**Figure 5:**
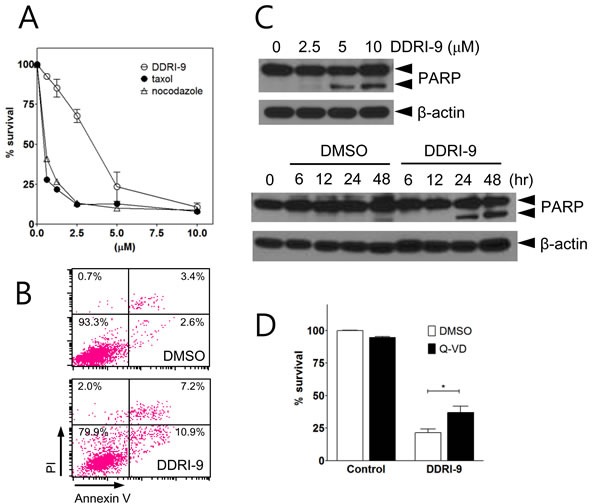
DDRI-9 induces cell death **A.** U2OS cells were treated with the indicated concentrations of DDRI-9 and mitotic inhibitors for 48 h, after which cell viability was evaluated using the MTT assay. Values represent the means ± SEM from three independent experiments. **B.** U2OS cells were incubated in 5 μM DDRI-9 for 24 h. Apoptotic cells were detected by flow cytometry after annexin V-FITC and PI staining. **C.** Protein extracts from U2OS cells treated with indicated concentrations of DDRI-9 for 24 h (upper) and 5 μM DDRI-9 for the indicated times were analyzed by Western blotting using antibodies against PARP-1 and β-actin. The proform of PARP (116 kDa) and cleaved PARP (85 kDa) are indicated. **D.** U2OS cells were pretreated with 5 μM Q-VD-OPh for 1 h before treatment with 5 μM DDRI-9 in DMEM containing 2% FBS. After 48 h, cell viability was evaluated using the MTT assay. The graphs and values represent the means ± SEM from three independent experiments (Student's t-test, (*) *P* < 0.05).

Because antimitotic drugs induce cell death by interfering with mitotic spindle microtubule dynamics in proliferating cells, they have been used to target proliferating tumor cells. To determine whether DDRI-9 could induce cytotoxicity, MTT assays in which U2OS cells were exposed to a serial dose of DDRI-9 or antimitotic drugs (taxol and nocodazole) for 48 h were performed. DDRI-9 alone induced U2OS cell death but was less cytotoxic than the antimitotic drugs (Figure 5A). In addition, we observed DDRI-9 cytotoxicity in various cell lines (HeLa, HCT116, HT29, MDA-MB-231, MCF-7, SK-BR3, and A549 cells), with varying LD_50_ values (Supplementary Table 1).

We next investigated whether DDRI-9-induced cell death was due to apoptosis. We evaluated markers of apoptosis (annexin V-positive cells and PARP cleavage). Based on flow cytometric analysis, the proportion of cells that were annexin V-positive increased in DDRI-9-treated U2OS cells compared to DMSO-treated cells (from 6.0% in DMSO vehicle-treated cells to 18.1% in DDRI-9-treated cells) (Figure 5B). Cleaved PARP was detected in DDRI-9-treated cells by Western blotting (Figure 5C). To confirm whether DDRI-9-induced cell death was due to caspase-dependent apoptosis, we pretreated U2OS cells with the pan-caspase inhibitor Q-Val-Asp-OPh (Q-VD-Oph) prior to treatment with DDRI-9. In the presence of Q-VD-OPh, DDRI-9-induced cell death decreased significantly (Figure 5D). DDRI-9 was also capable of inducing cell death in HeLa cells (Supplementary Figure 5). These data indicated that DDRI-9 alone could induce tumor cell death, which could be partially attributed to apoptosis.

## DISCUSSION

We previously developed a cell-based high content screening method using γH2AX foci quantitation to identify DDR inhibitors [25]. A novel DDR inhibitor, DDRI-18, was identified that delayed resolution of γH2AX foci, inhibited the DDR, and potentiated the cytotoxicity of DNA-damaging agents [27]. In this study, we characterized DDRI-9, another novel DDR inhibitor, and found that it competently inhibited γH2AX foci formation and delayed DNA repair processes after DSBs. In addition, DDRI-9 potentiated the cytotoxicity of ETO, a DNA damage-inducing agent. These results indicated that DDRI-9 could act as a typical DDR inhibitor, similar to the ATM kinase inhibitor KU55933 (Figures 1 and 3). However, DDRI-9 exerted slightly different inhibitory effects than KU55933 on the recruitment of DDR-related proteins to DNA damage sites. Following DSBs, foci formation by most DDR-related proteins examined (γH2AX, ATM, 53BP1, BRCA1, and DNA-PK) was inhibited by DDRI-9 treatment, similar to the effects of KU55933 treatment. However, foci formation by MDC1 increased after DDRI-9 treatment (Figure 2A). MDC1 functions as a platform for the recruitment of DDR-related proteins and amplifies ATM-dependent DDR signaling [17]. Recruitment of MDC1 to sites of DNA damage was concomitant with the recruitment of the DDR-related proteins. Thus, the opposite effects of DDRI-9 after DNA DSBs (a reduction in the foci formation of DDR-related proteins and an increase in the foci formation of MDC1) suggest that DDRI-9 could abrogate the ATM-dependent DDR signaling pathway at or downstream of MDC1.

We hypothesized that DDRI-9 binding might induce a conformational change in MDC1 that facilitates recruitment onto sites of DNA damage independent of γH2AX. A conformational change in MDC1 could prevent binding to phospho-ATM and result in a failure of amplification of ATM-dependent DDR signaling. In light of this hypothesis, it was interesting that DDRI-9 blocked mitotic progression (Figure 4), because a previous report indicated that MDC1 regulated mitotic progression [21]. In this study, siRNA knockdown of MDC1 caused mitotic arrest in HeLa cells. DDRI-9 may block mitotic progression by inhibiting the mitotic functions of MDC1. We therefore hypothesized that DDRI-9 targeted MDC1 or factors downstream of MDC1 in DDR signaling pathways. To test our hypothesis, we evaluated the inhibitory activity of DDRI-9 in MDC1-depleted cells. We found that depletion of MDC1 by siRNA resulted in inhibition of DDR (decreased γH2AX foci formation) and blockage of mitotic progression (increased mitotic cells) as previously reported. However, DDRI-9 treatment of MDC1-depleted cells did not result in additive inhibitory effects (data not shown). This raised the possibility that MDC1 was a target of DDRI-9.

To biochemically define the target using biotinylated DDRI-9, we attempted to determine the structure-activity relationship of DDRI-9 and identify the possible biotin-linking sites. However, all 215 derivatives tested (provided by the Korea Chemical Bank) failed to show inhibitory activity toward the DDR and mitotic progression. Thus, we could not determine a proper biotin-linking site for DDRI-9. We also performed co-immunoprecipitation experiments to identify the targets of DDRI-9. Lysates from DDRI-9-treated cells were immunoprecipitated with specific antibodies against candidate DDRI-9 targets such as MDC1 and ATM, and the presence of DDRI-9 analyzed by mass spectrometry. DDRI-9 was not detected by liquid chromatography-mass spectrometry or matrix-assisted laser desorption/ionization time of flight mass spectrometry, possibly due to the poor ionization of the compound or changes in the chemical structure. We were therefore unable to confirm that MDC1 was a target of DDRI-9.

The majority of DNA-damaging agents induce tumor cell death through apoptosis. Cell death induced by DDRI-9 was due to apoptosis because it was accompanied by phosphatidylserine (PS) exposure (an increase in annexin V-positive cells) and increased PARP cleavage, which are hallmarks of apoptosis. Inhibition of cell death by treatment with Q-VD-OPh revealed that DDRI-9-induced cell death was caspase-dependent. It is not likely that apoptotic cell death is the sole mechanism of DDRI-9-induced cell death, because annexin V-positive cells and cleaved PARP were not significantly increased, and most populations of DDRI-9-treated cells could not be recovered after treatment with the pan-caspase inhibitor Q-VD-OPh (Figure 5). We therefore examined whether DDRI also induced cell death through non-apoptotic mechanisms. Indeed, DDRI-9 treatment increased LC3 II conversion and LC3 puncta (autophagosomes), which are hallmarks of autophagy [28]. However, pharmacological inhibition of autophagy with 3-methylamine and chloroquine did not inhibit DDRI-9-induced cell death in U2OS cells (data not shown). Additionally, treatment with necrostatin, an inhibitor of necroptosis, did not inhibit DDRI-9-induced cell death (data not shown). These data indicated that DDRI-9-induced cell death was due to apoptosis and not autophagy or necroptosis. It is possible that mitotic catastrophe induction is the major mechanism underlying DDRI-9-induced cell death. This is because DDRI-9 induces mitotic arrest and mitotic catastrophe is considered to be cell death initiated by aberrant mitosis [29]. However, whether mitotic catastrophe should be categorized as a *bona fide* cell death mechanism is controversial [30].

In conclusion, we identified a novel chemical compound, DDRI-9, that inhibits both the DDR and mitosis. This compound not only potentiated the efficacy of anticancer drugs, but also induced tumor cell death. The exact mechanisms of action of DDRI-9 are not yet clear. Thus, further studies are required to enhance our understanding of the connection between the DDR and mechanisms of mitotic arrest. Ultimately, this could lead to the development of novel anticancer drugs. Recent studies have emphasized the close relationship between DDR and mitotic checkpoint mechanisms. For example, ATM is activated during mitosis by Aurora B kinase and phosphorylates the mitotic checkpoint serine/threonine protein kinase Bub1, resulting in the inhibition of the anaphase-promoting complex and regulation of mitotic progression into anaphase [31]. Characterization of the mechanisms of action of DDRI-9 may reveal a novel therapeutic target.

## MATERIALS AND METHODS

### Cell culture

U2OS, HeLa, MDA-MB-231, and HCT116 cells were cultured in Dulbecco's Modified Eagle Medium (DMEM) supplemented with 10% fetal bovine serum (FBS; Hyclone, South Logan, UT, USA) and 2 mM glutamine at 37°C in a 5% CO_2_ atmosphere.

### Materials and antibodies

DDRI-9 (C_13_H_10_FN_5_O_4_) was provided by the Korea Chemical Bank (Daejeon, Korea) or purchased from either ChemDiv (La Jolla, CA, USA) or Hanchem (Daejeon, Korea). KU55933 (C_21_H_17_NO_3_S_2_) was purchased from Calbiochem (San Diego, CA, USA). ETO was purchased from Sigma-Aldrich (St. Louis, MO, USA) and Q-VD-OPh was purchased from Tocris (Bristol, UK). The anti-γH2AX (Ser139) and phospho-ATM (Ser1981) antibodies were purchased from Millipore (Temecula, CA, USA). The anti-MDC1, 53BP1, NBS1, H2AX, and phospho-BRCA1 (Ser1423) antibodies were purchased from Bethyl Laboratories (Montgomery, TX, USA). The anti-phospho-DNA-PK antibody (Thr2609) and anti-BRCA1 antibody were purchased from Abcam (Cambridge, UK) and NeoMarkers (Fremont, CA, USA), respectively. The anti-phospho-CHK2 (Thr68), NBS1 (Ser343), CHK2, and Aurora A antibodies were purchased from Cell Signaling Technology (Danvers, MA, USA). Unless otherwise specified, all other chemicals and reagents were purchased from Sigma-Aldrich (St. Louis, MO, USA).

### Immunofluorescence

One day prior to treatment, cells were seeded at a density of (1 × 10^5^ cells per mL) in black 96-well plates with clear flat bottoms (Costar, Corning, NY, USA) or on cover slips in a 12-well plate. After chemical treatment, cells were rinsed with PBS, fixed with 3.7% formaldehyde in PBS for 20 min at room temperature (RT), and then washed three times with PBS for 5 min each. Non-specific binding was blocked by incubating the cells with 1% BSA and 0.02% Triton X-100 in PBS for 30 min at RT. The cells were sequentially incubated at RT with the primary antibodies (1:500 dilutions) for 3 h, Alexa Fluor^®^ 488-conjugated anti-rabbit or mouse immunoglobulin G antibodies (1:500, Molecular Probes, Carlsbad, CA, USA) for 1 h, and Hoechst 33342 (10 μg/mL, Molecular Probes) for 10 min. The cells were washed three times with 0.02% Triton X-100 in PBS for 10 min after each incubation and were visualized using an IN Cell Analyzer 1000 (GE Healthcare, Buckinghamshire, UK) or confocal microscope (Carl Zeiss Microscopy GmbH, Jena, Germany).

### Image acquisition and analysis

The total area of γH2AX, phospho-ATM, MDC1, 53BP1, phospho-BRCA1, and phospho-DNA-PK foci was measured using an IN Cell Analyzer 1000. Images of stained cells were acquired from the automated fluorescence microscope platform of the IN Cell Analyzer 1000 using a 20 × objective lens. Images from more than five fields per well were collected to obtain data for 200**-**400 cells. The filter sets used for detection of the Hoechst 33342 and Alexa Fluor^®^ 488 signals were D360/40 (excitation)-HQ535/50 (emission) and D475/20 (excitation)-HQ 535/20 (emission), respectively. The images were analyzed using the Multi Target Analysis (MTA) module of the IN Cell Analyzer 1000 Workstation software (v3.4). The MTA module measured the fluorescence intensity and area (μm^2^) of nuclei (based on Hoechst 33342 staining) and targeted foci (based on Alexa Fluor^®^ 488 staining). The fluorescence area of Alexa Fluor^®^ 488 represented γH2AX foci. We previously reported that the foci total area per cell was well correlated with the extent of DNA damage [25, 27].

### Comet assay

The comet assay was performed under alkaline conditions according to the Trevigen CometAssay^®^ kit manual (Gaithersburg, MD, USA). Cells treated with chemical compounds and ETO and were harvested at the indicated time points. A total of 5 × 10^2^ cells in 5 μL of PBS was mixed with 50 μL of low melting agarose, placed onto a CometSlide^™^, solidified at 4°C for 10 min, immersed in lysis solution at 4°C for 40 min, and electrophoresed at 21 volts for 30 min in alkaline buffer (300 mM NaOH, 1 mM EDTA) in the dark. The slides were then immersed in distilled water followed by 70% ethanol and then dried. DNA was stained with ethidium bromide and analyzed using KOMET 5.5 software (Kinetic Imaging, Ltd., Nottingham, UK).

### Clonogenic assay

Cells were seeded at a density of 100**-**200 cells per well in a six-well plate and exposed to anticancer drugs and DDRI-9 at the indicated concentrations for 48 h. The cells were then incubated in drug-free medium for 7**-**10 days until colonies (50 cells per colony) were visualized. Following incubation, colonies were fixed and stained with Diff-Quick (Sysmex, Kobe, Japan).

### Western blotting

Cell lysates were prepared by suspending 4 × 10^5^ cells in 100 μL of Tris-Glycine SDS sample buffer containing 5% 2-mercaptoethanol (Novex, Carlsbad, CA, USA). The cell lysates were incubated on ice for 10 min and then boiled at 95°C for 5 min. Equal amounts of total protein were separated on a 4**-**12% gradient or a 10% or 15% Tris-Glycine PAGE and then transferred to Immobilon-P membranes (Millipore, Billerica, MA, USA). After blocking with 5% skim milk in TBST [20 mM Tris (pH 7.5), 135 mM NaCl, and 0.05% Tween 20], the membranes were incubated with the indicated primary antibodies, washed with TBST, and then incubated with horseradish peroxidase-conjugated anti-rabbit or anti-mouse IgG antibodies (1:5000 dilutions; Cell Signaling Technology, Danvers, MA, USA). After extensive washing with TBST, the proteins were visualized by chemiluminescence using the ECL reagent (GE Healthcare, Buckinghamshire, UK).

### MTT assay

Cells were seeded at a density of 5 × 10^3^ cells per well in 96-well plates and treated with taxol, nocodazole, or DDRI-9 at the indicated concentrations. After incubation for the indicated times, the cells were washed with PBS and 100 μL of MTT [3-(4,5-dimethylthiazol-2-yl)-2,5-diphenyl tetrazolium bromide, 0.5 mg/mL] was added. After incubation at 37°C for 2 h in a 5% CO_2_ environment, insoluble crystals were completely dissolved in DMSO. The absorbance at 540 nm was measured using a VersaMax^™^ Microplate Reader (Molecular Devices, Sunnyvale, CA, USA).

### Detection of mitotic cells using an anti-phospho-histone H3 (Ser10) antibody

Cells were fixed with 1% formalin on ice for 15 min, washed with PBS, and then fixed with cold 70% ethanol at −20°C for at least 30 min. After fixation, the cells were washed with PBS and permeabilized with 0.25% TritonX-100 in PBS on ice for 5 min. The cells were sequentially incubated at RT with 1 μg of anti-phospho-histone H3 (Ser10; Abcam, Cambridge, UK) for 1 h, Alexa Fluor^®^ 488-conjugated anti-mouse IgG antibody (Molecular Probes, Carlsbad, CA, USA) for 30 min, and propidium iodide (PI, 10 μg/mL) for 20 min at RT in the dark. Finally, the cells were analyzed using FACSCaliber^™^ (BD Biosciences, San Jose, CA, USA).

### Statistical analysis

Data are presented as the mean values ± SEM. All statistical analyses were performed with GraphPad Prism^®^ version 5.03 (GraphPad Software, San Diego, CA, USA). Comparisons between two groups were performed using the Student's *t*-test for unpaired data. Differences between groups were considered statistically significant if *P* < 0.05.

## SUPPLEMENTARY FIGURES AND TABLES


