# Supplementary material for: DDRI-9: a novel DNA damage response inhibitor that blocks mitotic progression
Source: Oncotarget. 2016 Feb 2;7(14):17699–710. doi: 10.18632/oncotarget.7135 (PMC4951243; doi:10.18632/oncotarget.7135)
Supplement: Supplementary file 1 [file oncotarget-07-17699-s001.pdf]

DDRI-9: a novel DNA damage response inhibitor that blocks mitotic progression

Supplementary Material

Supplementary Table 1

LD_{50} of DDRI-9 in various cell lines.

The indicated cell lines were incubated with various concentrations of DDRI-9 for 48 h. Cell viability was then evaluated using the MTT assay. LD_{50} values were calculated with GraphPad Prism version 5.03 (GraphPad Software, San Diego, CA).

	LD50 of DDRI-9 (μM)
	Ave ± SD
**U2OS**	3.02 ± 0.39
**HeLa**	5.74 ± 0.32
**HCT116**	4.92 ± 2.92
**HT29**	3.15 ± 0.84
**MDA-MB-231**	1.81 ± 0.05
**MCF7**	2.19 ± 0.62
**SK-BR3**	5.17 ± 0.99
**A549**	15.41 ± 7.95

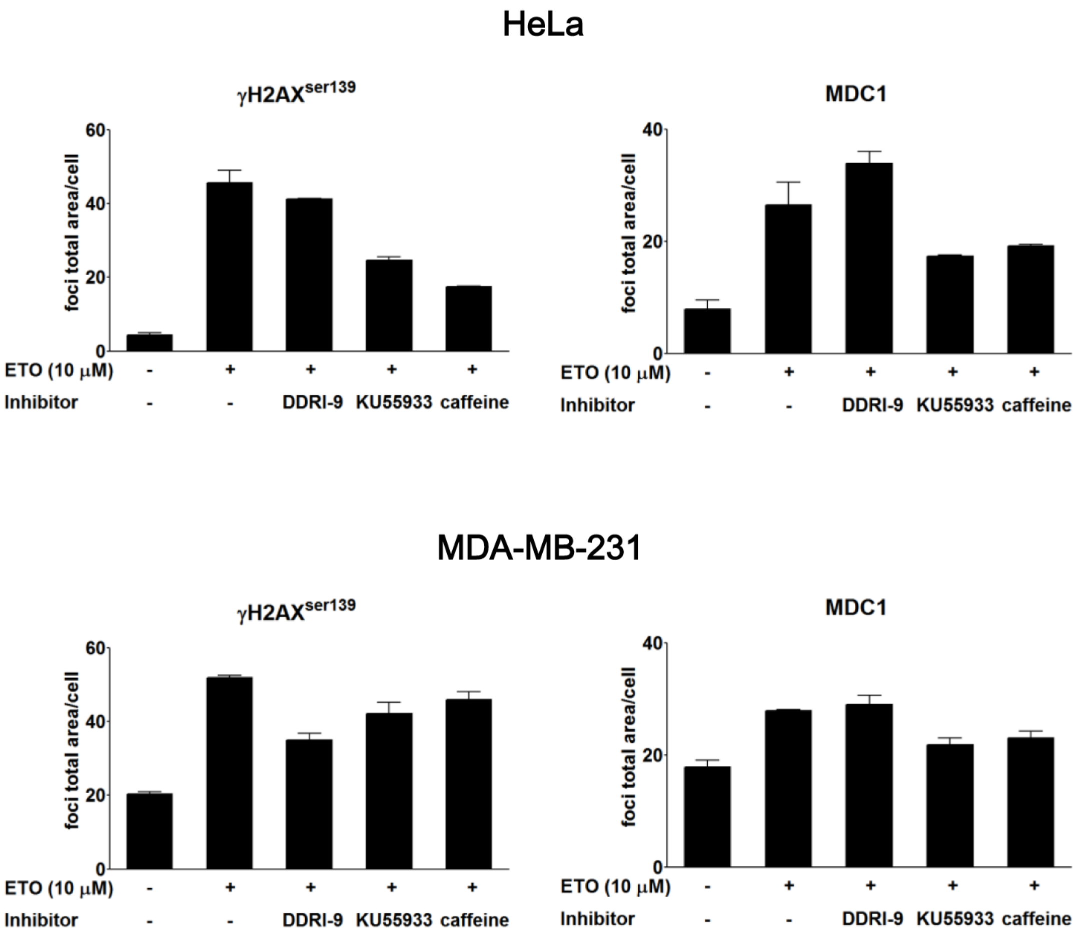

Supplementary Figure 1: **DDRI-9 inhibited γH2AX foci formation in HeLa and MDA-MB-231 cells.**

HeLa and MDA-MB-231 cells were pretreated with 2.5 μM DDRI-9, 10 μM KU55933 and 10 mM caffeine for 1 h and then exposed to 10 μM ETO for 1 h. After incubation, the cells were fixed and processed for immunofluorescence with the indicated antibodies, and the foci were analyzed with an IN Cell Analyzer. Representative graphs and values from three independent experiments are shown. Values represent the means ± SEM.

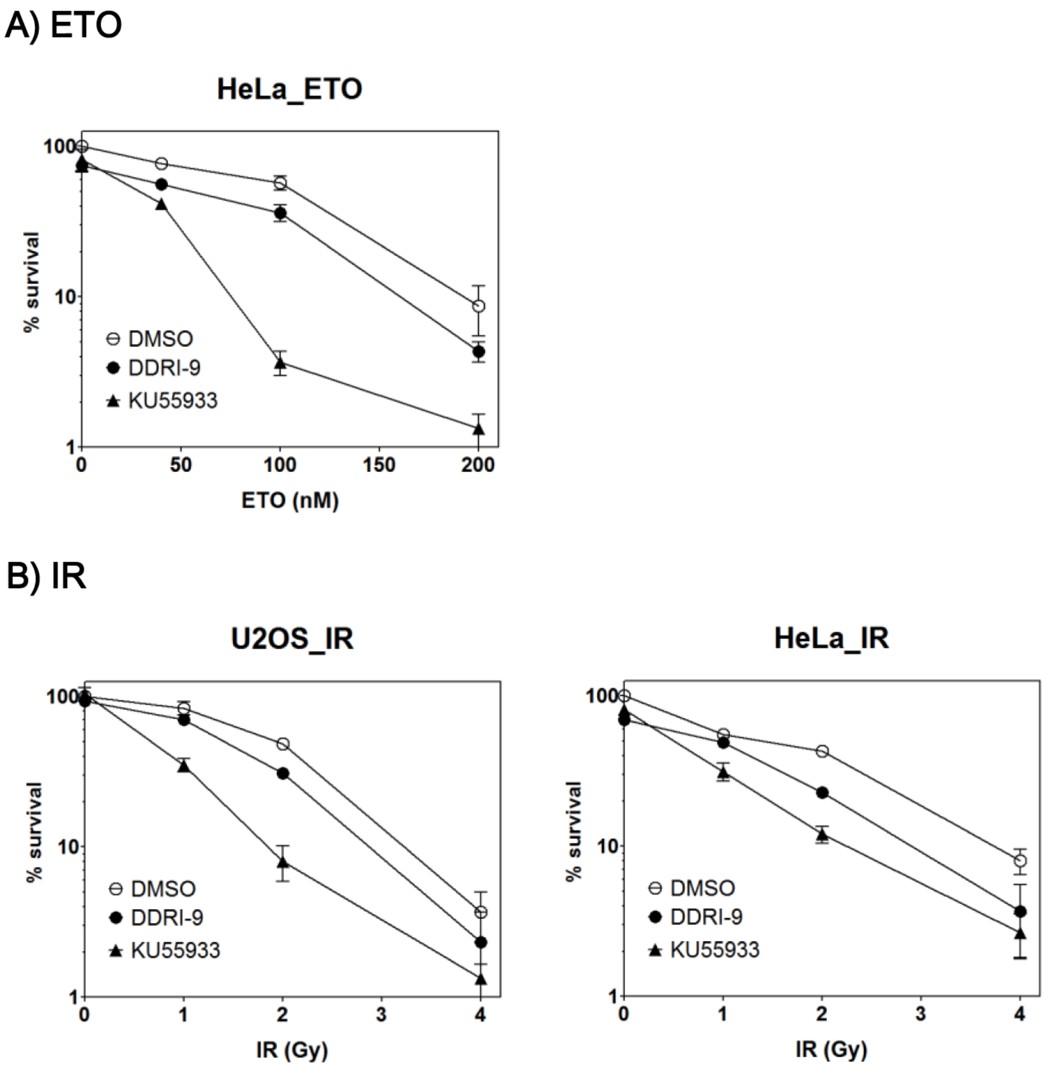

Supplementary Figure 2: **DDRI-9 potentiated cytotoxicity of DNA damaging agents in U2OS and HeLa.**

A.HeLa cells were pretreated with 0.1 μM DDRI-9 or 5 μM KU55933 for 1 h and then incubated with ETO at the indicated concentrations for 48 h, followed by incubation in ETO- and chemical-free medium until colony formation. Survival was measured by colony counting. Representative graphs from three independent experiments are shown. Values represent the means ± SEM.

B.U2OS cells and HeLa cells were pretreated with 0.1 μM DDRI-9 and 5 μM KU55933 for 1 h and then exposed to ionizing radiation at indicated Gy. Forty-eight hours after exposure, the media was changed to chemical-free media and the cells were incubated until colony formation. Survival was measured as above (A).

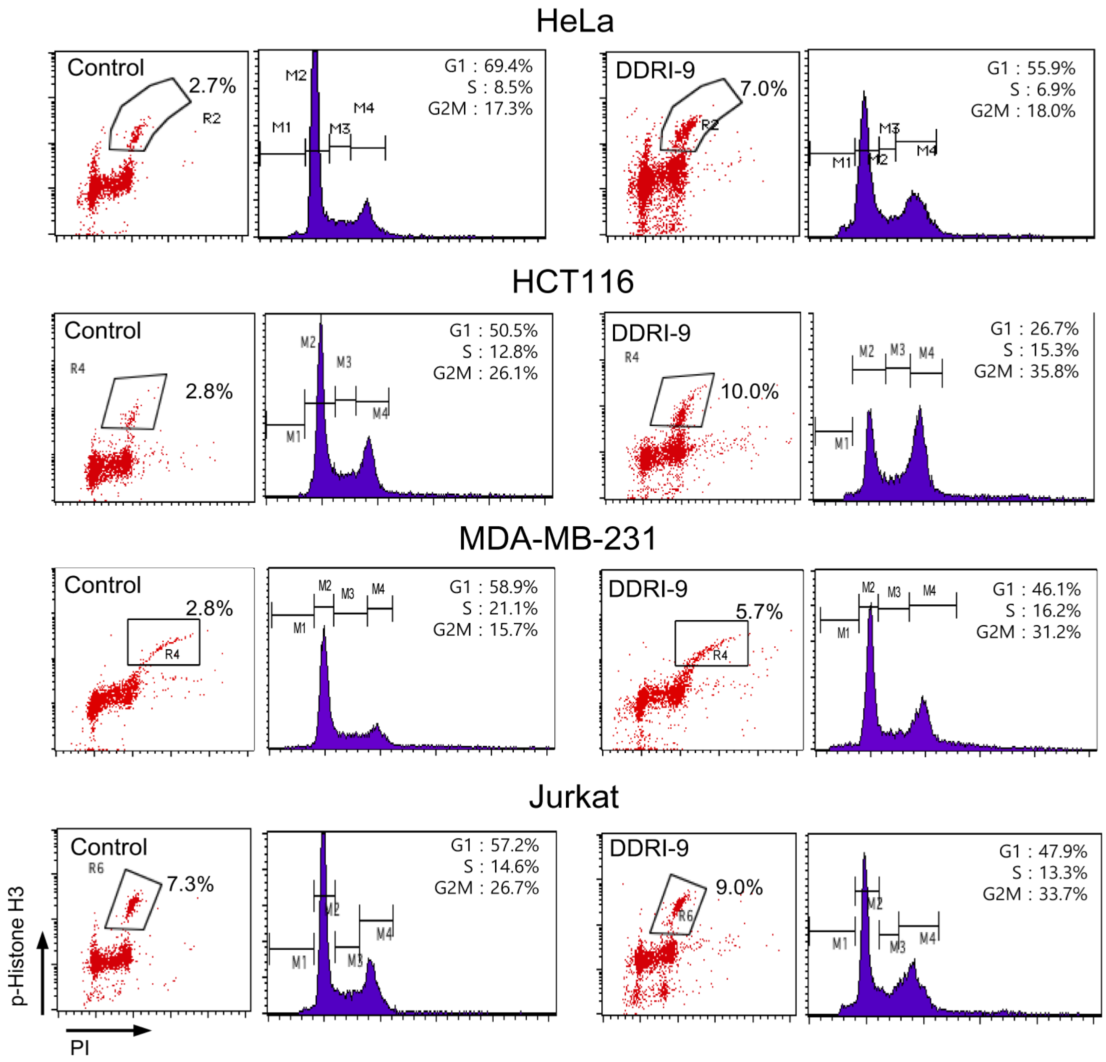

Supplementary Figure 3: **DDRI-9 blocks mitotic progress in various cell lines.**

The indicated cell lines were incubated with 2.5 μM DDRI-9 for 24 h. After incubation, the cells were fixed and processed for immunofluorescence with an anti-phospho-histone H3 antibody and PI, and analyzed by flow cytometry.

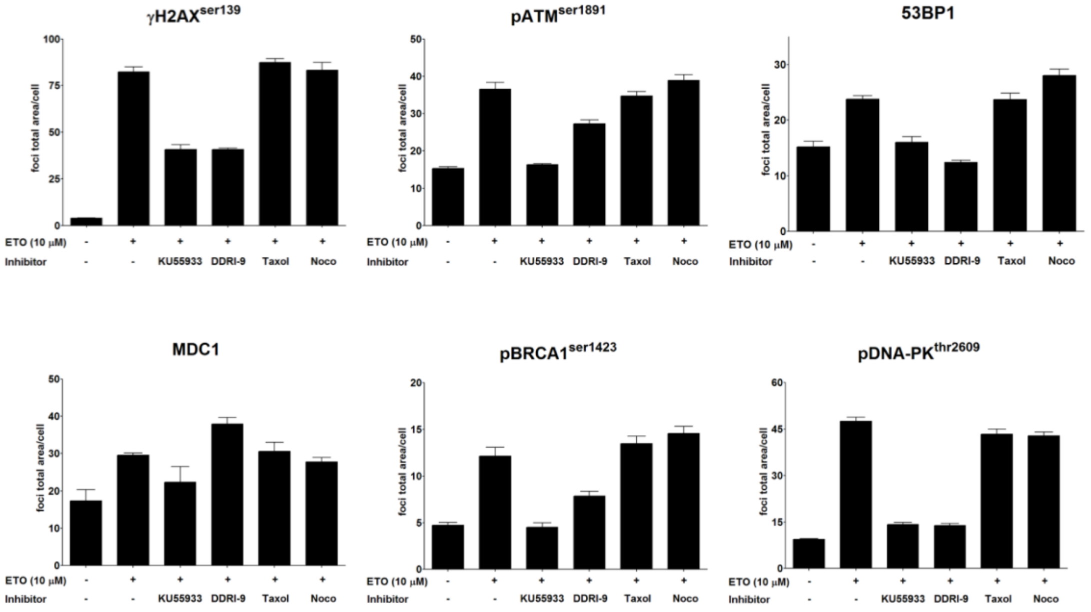

Supplementary Figure 4: **Mitotic inhibitors do not affect the recruitment of DDR-related proteins.** U2OS cells were pretreated with 2.5 μM DDRI-9, 10 μM KU55933, 100 nM taxol and 200 nM nocodazole for 1 h and then exposed to 10 μM ETO for 1 h. After incubation, the cells were fixed and processed for immunofluorescence with indicated antibodies, and the foci were analyzed with an IN Cell Analyzer. Representative graphs and values from three independent experiments are shown. Values represent the means ± SEM.

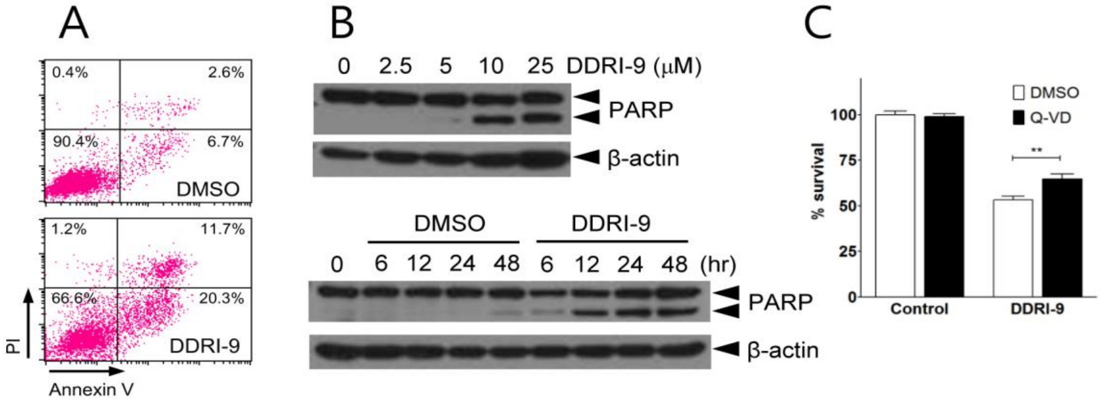

Supplementary Figure 5: **DDRI-9 induced cell death through apoptosis in HeLa cells.**

A.HeLa cells were incubated in 5 μM DDRI-9 for 24 h. Apoptotic cells were detected by flow cytometry after annexin V-FITC and PI staining.

B.Protein extracts from HeLa cells treated with the indicated concentrations of DDRI-9 for 24 h (upper), and with 10 μM DDRI-9 for the indicated times (down) were analyzed by Western blotting using antibodies against PARP-1 and β-actin. The proform of PARP (116 kDa) and cleaved PARP (85 kDa) are indicated.

C.HeLa cells were pretreated with 5 μM Q-VD-OPh for 1 h before treatment with 5 μM DDRI-9. After 48 h, cell viability was evaluated using the MTT assay. Graphs and values represent the means ± SEM from three independent experiments (Students *t*-test, (**) $P < 0.01$).
